# Neonatal Screening for Congenital Adrenal Hyperplasia in Turkey: Outcomes of Extended Pilot Study in 241,083 Infants

**DOI:** 10.4274/jcrpe.galenos.2020.2019.0182

**Published:** 2020-09-02

**Authors:** Tülay Güran, Başak Tezel, Meltem Çakır, Ayşehan Akıncı, Zerrin Orbak, Mehmet Keskin, Beray Selver Eklioğlu, Alev Ozon, Mehmet Nuri Özbek, Gülay Karagüzel, Nihal Hatipoğlu, Fatih Gürbüz, Filiz Mine Çizmecioğlu, Cengiz Kara, Enver Şimşek, Firdevs Baş, Murat Aydın, Feyza Darendeliler

**Affiliations:** 1 Marmara University Faculty of Medicine, Department of Paediatric Endocrinology and Diabetes, İstanbul, Turkey; 2 Turkish Directorate of Public Health, Ankara, Turkey; 3 Mersin City Hospital, Clinic of Paediatric Endocrinology and Diabetes, Mersin, Turkey; 4 İnönü University Faculty of Medicine, Department of Paediatric Endocrinology and Diabetes, Malatya, Turkey; 5 Atatürk University Faculty of Medicine, Department of Paediatric Endocrinology and Diabetes, Erzurum, Turkey; 6 Gaziantep University Faculty of Medicine, Department of Paediatric Endocrinology and Diabetes, Gaziantep, Turkey; 7 Necmettin Erbakan University, Meram Faculty of Medicine, Department of Paediatric Endocrinology and Diabetes, Konya, Turkey; 8 Hacettepe University Faculty of Medicine, Department of Paediatric Endocrinology and Diabetes, Ankara, Turkey; 9 Gazi Yaşargil Training and Research Hospital, Department of Paediatric Endocrinology and Diabetes, Diyarbakır, Turkey; 10 Karadeniz Techical University Faculty of Medicine, Department of Paediatric Endocrinology and Diabetes, Trabzon, Turkey; 11 Erciyes University Faculty of Medicine, Department of Paediatric Endocrinology and Diabetes, Kayseri, Turkey; 12 Çukurova University Faculty of Medicine, Department of Paediatric Endocrinology and Diabetes, Adana, Turkey; 13 Kocaeli University Faculty of Medicine, Department of Paediatric Endocrinology and Diabetes, Kocaeli, Turkey; 14 Ondokuz Mayıs University Faculty of Medicine, Department of Paediatric Endocrinology and Diabetes, Samsun, Turkey; 15 Osmangazi University Faculty of Medicine, Department of Paediatric Endocrinology and Diabetes, Eskişehir, Turkey; 16 İstanbul University İstanbul Faculty of Medicine, Department of Paediatric Endocrinology, İstanbul, Turkey

**Keywords:** Neonatal screening, congenital adrenal hyperplasia, second-tier, steroid profiling, incidence, 11β-hydroxylase deficiency

## Abstract

**Objective:**

Turkish Directorate of Public Health introduced the first pilot screening program for congenital adrenal hyperplasia (CAH) in four Turkish cities in 2017, and in 2018 extended the program, with a slight change in screening strategy, to fourteen cities. To evaluate the performance of the extended study and update previously reported outcomes.

**Methods:**

Retrospective, descriptive study. Neonates of ≥32 gestational weeks and ≥1500 gr birth weight from fourteen cities, born between May-December 2018, were included. Screening protocol included one sample, two-tier testing as applied in the previous pilot study. In the first step, 17α-hydroxyprogesterone (17-OHP) was measured by fluoroimmunoassay in dried blood spots (DBS) obtained at 3-5 days of life. Cases with positive initial screening underwent second tier testing by steroid profiling in DBS using liquid chromatography-tandem mass spectrometry to measure 17-OHP, 21-deoxycortisol (21-S), cortisol (F), 11-deoxycortisol and androstenedione. The babies with a steroid ratio (21-S+17-OHP)/F of ≥0.7 (increased from ≥0.5 in the earlier pilot study) were referred to pediatric endocrinology clinics for diagnostic assessment.

**Results:**

In the evaluated period, 241,083 newborns were screened. 12,321 (5.11%) required second-tier testing and 880 (0.36%) were referred for clinical assessment, twenty of whom were diagnosed with CAH (10 females, 10 males). Sixteen were diagnosed as classical 21-hydroxylase deficiency (21-OHD) CAH (12 with salt-wasting and four with simple virilising CAH), and four cases were identified with 11β-OHD CAH. No case of salt-wasting CAH was missed by neonatal screening (sensitivity was 100%). The incidence of classical 21-OHD and 11β-OHD in the screened population was 1:15,067 and 1:60,270, respectively.

**Conclusion:**

Turkish neonatal CAH screening effectively led to earlier diagnosis of 21-OHD and 11β-OHD, using steroid profiling as a second-tier test. This will result in improved care of these patients in the future.

**What is already known on this topic?**

Classical congenital adrenal hyperplasia (CAH) occurs in 1:13,000 to 1:15,000 live births. 21-hydroxylase enzyme deficiency (21-OHD) occurs in 90 to 95% of all cases of CAH. In contrast to 21-OHD, the carrier rate for 11β-OHD is fairly low, the rate of compound heterozygosity of *CYP11B1* mutations in the pathogenesis is less frequent and the estimated overall frequency is 1 in 100,000 live births. However, the prevalence of 11β-OHD is relatively higher in the Middle East and North Africa. Neonatal screening for CAH is effective in detecting the salt-wasting form and thereby reducing mortality. The estimated incidence of classical 21-OHD was 1:7,787 in an initial pilot study of newborn screening for CAH, in which 38,935 neonates were screened in Turkey.

**What this study adds?**

The incidence of classical 21-OHD and 11β-OHD CAH in Turkey in the screened population of 241,083 neonates was 1:15,067 and 1:60,270, respectively. Turkish neonatal CAH screening led to the early and effective diagnosis of 21-OHD and 11β-OHD by the use of steroid profiling as a second-tier test.

## Introduction

The most common cause of congenital adrenal hyperplasia (CAH) is 21-hydroxylase deficiency (21-OHD), which accounts for about 95% of CAH in most populations. Severe deficiency of the 21-hydroxylase enzyme leads to life-threatening adrenocortical insufficiency in both sexes and varying degrees of pathology of the external genitalia in females, and it is associated with high mortality during the first days of life and increased risk for incorrect sex assignment. Neonatal screening for 21-OHD CAH is effective in detecting the severe forms (classical 21-OHD) and reducing mortality and is helpful in correct sex assignment of female cases. To this end, the Turkish Directorate of Public Health (TDPH) introduced the first Turkish newborn screening (NBS) programme for CAH in 2017 in four Turkish cities as a pilot study. The incidence of classical 21-OHD in the screened population was estimated at 1:7,787, which was high compared to many other countries (1,2). However, this incidence figure required validation in a larger number of babies from nationwide screening.

The NBS programme for CAH adopted a one sample, two-tier testing protocol in the 2017 pilot. This resulted in a recall rate for clinical assessment of 0.54% after a positive second tier test. In the pilot study the second tier test consisted of measuring 17-hydroxyprogesterone (17-OHP), 21-deoxycortisol (21-S), cortisol (F), 11-deoxycortisol and androstenedione using liquid chromatography-tandem mass spectrometry (LC-MS/MS). The ratio of (21-S+17-OHP)/F was calculated and a cut-off value for this ratio of ≥0.5 resulted in referral to pediatric endocrinology clinics for formal assessment. In 2018, the TDPH extended the screening the programme to fourteen cities including a slightly higher second-tier cut-off of ≥0.7 in screening strategy in order to decrease recall rate and cost of screening without missing classical 21-OHD cases.

Although 11β-OHD is the second most common cause of CAH (5-8%) with an incidence of about 1:100,000, in 2017 one male 11β-OHD CAH case was identified among 38,935 neonates screened. The prevalence of 11β-OHD is reported to be relatively high in the Middle East and North Africa (3). An advantage of the Turkish second-tier screening protocol is that it includes 11-deoxycortisol measurement which is a diagnostic steroid for 11β-OHD CAH, in addition to 17-OHP, 21-S, cortisol and androstenedione. Hence, the adopted second-tier CAH screening strategy would facilitate identification of 11β-OHD cases in addition to those with 21-OHD.

In this study, the results of the Turkish extended NBS programme for CAH were evaluated and the previous outcomes of the earlier pilot study have been updated.

## Methods

**Figure 1:**
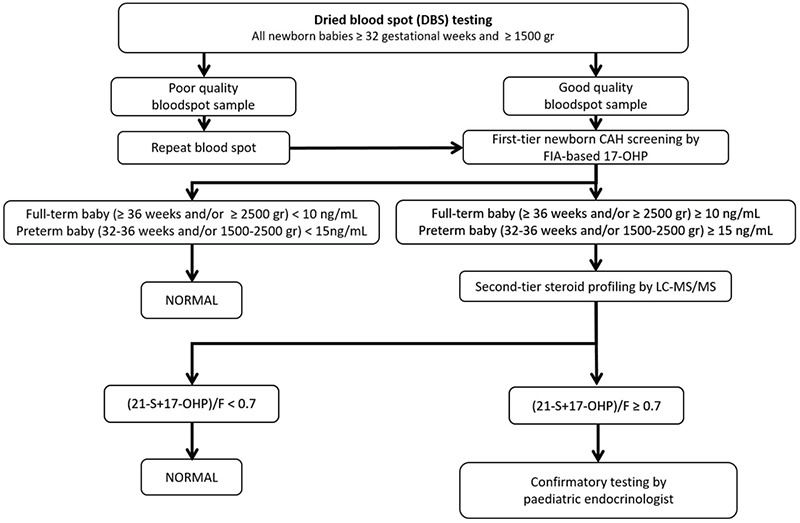
Flowchart for extended neonatal congenital adrenal hyperplasia screening initiated by the Turkish Directorate of Public Health [17-hydroxyprogesterone (17-OHP) conversion factor from ng/mL to nmol/L: multiply by 3.02] CAH: congenital adrenal hyperplasia, FIA: fluoroimmunoassay, LC-MS/MS: liquid chromatography-tandem mass spectrometry, 17-OHP: 17-hydroxyprogesterone, 21-S: 21-deoxycortisol, F: cortisol

The extended screening programme for CAH occurred between March and December 2018, in fourteen cities (Konya, Adana, Kayseri, Samsun, Ankara, Gaziantep, Diyarbakır, Mersin, Kahramanmaraş, Elazığ, Erzurum, Malatya, Trabzon, and Van) as directed by TDPH. The CAH screening algorithm was the same as used in 2017 (1) with the exception of an increased steroid profile ratio cut-off for second-tier testing (Figure 1). Heel-prick blood samples were studied, as previously described (1). Initial CAH screening was based on the measurement of 17-OHP in dried blood spot (DBS) on filter paper by fluoroimmunoassay (Labsystems Diagnostics, Finland). Cut-off values for 17-OHP were based primarily on gestational age and birth weight. 17-OHP values of 10 ng/mL and 15 ng/mL have been used as cut-off points for newborn babies ≥36 gestational weeks (gw) and/or ≥2500 grammes (gr) birth weight, and for newborn babies between 32-36 gw and/or 1500-2500 gr birth weight, respectively. If the 17-OHP level was above the cut-off level in the first-tier test, the filter paper was directly analyzed by LC-MS/MS for a steroid profiling assay. This simultaneously analyzes 17-OHP, 21-deoxycortisol (21-S), cortisol (F), androstenedione (4AS) and 11-deoxycortisol (11-S). Normal values for babies 32-36 weeks and/or 1500-2500 gr were; 17-OHP: <8 ng/mL, 21-S: <1.5 ng/mL, F: >50 ng/mL, 4AS: <4.5 ng/mL and 11-S: <1.8 ng/mL. Normal ranges for babies ≥36 weeks and/or ≥2500 gr were; 17-OHP: <1.5 ng/mL, 21-S: <1.5 ng/mL, F: >50 ng/mL, 4AS: <4.5 ng/mL and 11-S: <1.8 ng/mL. Although all steroids were evaluated in each baby, a steroid ratio ≥0.7 and/or an elevation of 11-S (>10 ng/mL) were considered as the main criteria for referral for formal clinical evaluation for CAH.

Final calculations of true-positive (TP), false-positive (FP), true-negative (TN) and false-negative (FN) screening results were made. Efficiency of screening protocol was assessed with positive predictive value (PPV), sensitivity and specificity calculated by the following formulas: PPV=TP/(TP+FP); sensitivity=TP/(TP+FN); specifity=TN/(TN+FP).

### Ethics

The parents were informed about NBS. Heel-prick blood samples were collected from live-born babies after written consent from the parents was obtained. The study was carried out with the written permission of the Scientific Committee of the TDPH.

### Statistical Analysis

Statistical evaluation was performed using GraphPad Prism® V5.0 software (GraphPad Software Inc., San Diego, California, USA). The results for each steroid are reported as mean, standard deviation (SD) or as median (interquartile range) in the text. The 99.8% and 99.5% values of 17-OHP are shown for healthy babies in order to define healthy cut-off values.

## Results

A total of 241,083 neonates underwent CAH screening. Of those, 220,367 (91.4%) were ≥36 gw and ≥2500 gr birth weight. There were 16,919 babies (7.0%) between 1500-2500 gr birthweight and 11,017 babies (4.5%) were born between 32-36 gw. In addition 7,220 (2.9%) of the babies were born between 32-36 gw and had a birthweight of 1500-2500 gr.

Results of first-tier 17-OHP measurement in DBS from the healthy newborn population (those without CAH) are summarized in Table 1. The 99.8 and 99.5 percentile values for capillary 17-OHP concentration for healthy babies are shown in order to define healthy cut-off values with greater sensitivity (https://www.ncbi.nlm.nih.gov/pubmed/?term=Hayashi%20G%5BAuthor%5D&cauthor=true&cauthor_uid=22218447) (4).

In total 12,321 (5.1%) babies had second-tier testing by LC-MS/MS steroid profiling using a single DBS. During screening the majority of babies that failed to pass the first-tier screen were born between 32-36 gw and/or 1500-2500 gr birthweight and required second-tier testing in comparison to those with a birthweight of ≥2500 gr and/or gestational age ≥36 weeks (Table 2).

Eight hundred and eighty babies, who failed to pass second-tier testing were referred to paediatric endocrinology clinics for further evaluation, which corresponds to an overall recall rate of 0.36%.

Table 3 shows the distribution of second-tier testing values of babies referred for further analysis and results are summarized with respect to gestational age and birth weight. The highest proportion of babies requiring formal assessment had (21S+17-OHP)/F ratio of between 0.7-1.

An increased level of 17-OHP was observed in the first tier testing of cases with 11β-OHD. On second-tier testing the cases with elevated 11-S (>10 ng/mL) (5) were referred to clinics with the potential diagnosis of 11β-OHD. These cases were further evaluated for 11β-OHD after referral to pediatric endocrinologists.

Consequently, 20 babies were diagnosed with CAH (10 females, 10 males). Sixteen were diagnosed with 21-OHD CAH. Of these sixteen 12 cases had salt-wasting and four cases had simple virilising 21-OHD CAH and four cases were identified with 11β-OHD CAH. No patients with salt-wasting CAH were missed by neonatal screening and thus the sensitivity was 100%. The incidence of classical 21-OHD and 11β-OHD in the screened population was 1:15,067 and 1:60,270, respectively. None of these babies was premature and none had a low birth weight. The definitive diagnoses of CAH cases identified and screening results are presented in Table 4. The screening results of patient number 6 were not available, although the patient was registered as CAH and hospital files recorded that he was referred after a positive screening test. His genetic results were obtained from the molecular diagnostic laboratory and were consistent with salt-wasting 21-OHD. After hydrocortisone and fludrocortisone treatments were started, the patient was lost to clinical follow-up.

The mean±SD (range) duration from birth to clinical evaluation of abnormal second-tier screening results of the cases was 17.35±5.64 (7-54) days.

Overall PPV, sensitivity and specificity of the current screening protocol for 21-OHD CAH was calculated as 1.9%, 100% and 99.7%, respectively. There were no FN case (Table 5).

## Discussion

In this retrospective analysis of extended pilot study of neonatal CAH screening, the incidence in Turkey of 21-OHD and 11β-OHD was determined as 1:15,067 and 1:60,270, respectively. We have analysed the characteristics and efficacy of the extended NBS for CAH and revisited our previous outcomes to enhance screening performance for the upcoming nationwide NBS for CAH in Turkey.

The incidence of classical CAH is approximately 1:14,000 to 1:18,000 in most populations (2), which is similar to our data in this extended CAH screening programme. The incidence of classical 21-OHD was estimated to be 1:7,787 in the initial pilot study, in which 38,935 neonates were screened. The high prevalence of CAH was attributed to a high rate of consanguinity in that study. However, in retrospect the overestimation is more probably due to low number of neonates included in the pilot. In addition, the rate of consanguinity differs in different regions of Turkey. Although the carrier rate for classical 21-OHD is fairly stable (~2%) in the general population, the prevalence of classical 21-OHD may change with the rate of consanguinity. Therefore, we would be able to determine more accurate prevalence of classical CAH once a nationwide NBS for CAH is established in Turkey.

In contrast to 21-OHD, the carrier rate for 11β-OHD is fairly low, the rate of compound heterozygosity of CYP11B1 mutations in the pathogenesis is less frequent and estimated overall frequency is 1 in 100,000 live births (3). However, it has been reported that the prevalence of 11β-OHD is relatively higher in the Middle East and North Africa (3). In this study, the incidence of 11β-OHD was found to be 1:60,270, which is comparable to the approximate incidence of 1:40,000 in the earlier pilot study (1). Based on the more recent findings, real life data on the incidence of 11β-OHD in Turkey shows that the incidence is twice as common as the estimated overall frequency reported by Khattab et al (3) The diagnosis 11β-OHD CAH is generally delayed and can cause significant morbidities including arterial hypertension, precocious pseudopuberty, genital virilization and testicular adrenal rest tumors. Inclusion of 11-deoxycortisol in our second-tier CAH screening strategy, made it possible to identify cases with 11β-OHD in addition to cases with 21-OHD. Early diagnosis and treatment of these cases may reduce the morbidity.

There was no mortality due to unrecognized classical CAH among screened cohort in the extended NBS programme. However, one of the failings of the pilot study was the delay in recall of positive screening results. In the extended screening programme the mean±SD duration from birth to clinical evaluation of abnormal screening test results of false positive cases was reduced to 17.35±5.64 from 25.8±6.4 days in the pilot. Nevertheless and despite the fact that there has been no mortality to date, this reduction in time to clinical evaluation does not suggest improved safety for the screening program as a salt-wasting crisis may develop due to the long current recall time. As seen in our current and previous data, all cases with salt-wasting 21-OHD had significantly elevated concentrations of 17-OHP at the first step of screening (≥90 ng/mL in almost all cases). Therefore, we suggest that a remarkably high 17-OHP value on first-tier testing (90 ng/mL), which is strongly suggestive of 21-OHD CAH, should be sufficient to directly recall neonates with such elevated 17-OHP concentrations. This approach would reduce the time to formal assessment and may reduce the risk of mortality due to salt wasting crises, at least in cases with severe 21-OHD.

The recall rate during extended NBS was calculated and compared to the previous pilot study. By increasing the cut-off value for the (21-S+17-OHP)/F ratio from ≥0.5 to ≥0.7, it was possible to reduce the recall rate from 0.54% to 0.36%. Importantly, this change did not lead to any missed cases with salt-wasting 21-OHD or 11β-OHD in the screened population. Supporting our previous analysis, 493/880 babies (56%) had (21-S+17-OHP)/F ratio <1 while this ratio ranged between 10.7-42.3 in salt-wasting 21-OHD cases. The lowest ratio value observed was 1.11 in a patient with simple virilizing 21-OHD and only one 11β-OHD case had a ratio <1. Therefore, if 1 had been used as the cut-off for (21-S+17-OHP)/F ratio, the recall rate would decrease by 56% without missing any classical 21-OHD cases.

Over the past 20 years screening programs for CAH have reported variable results for recall rates ranging between 0.002-1.2%, for PPV ranging between 0.1-60% and for sensitivity ranging between 75-100% (5,6,7,8,9,10,11). Despite higher costs, recall rates are lower and PPVs are better with two-tier screening programs. Therefore, two-tier testing may be more appropriate, particularly in populations with high birth rates and/or high rates of consanguinity. Employing LC-MS/MS as second tier test and the use of cut-off values adjusted for gestational age and birth weight are other important measures to improve PPV (12).

### Study Limitations

The prevelance of 21-OHD and 11-OHD was estimated among approximately 240,000 babies screened in this pilot study. This figure may change and may need to be recalculated after nationwide NBS is established in Turkey.

## Conclusion

In conclusion, the current NBS strategy for CAH is efficient in identifying cases with both classical 21-OHD and 11β-OHD, which may allow for improved care of these patients and reduce morbidity in the future. However, further improvements to reduce recall time and the recall rate of abnormal screening test results is warranted.

## Figures and Tables

**Table 1:**
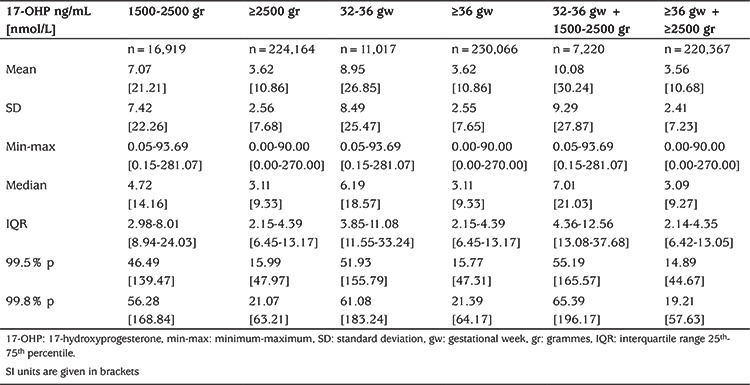
Fluoroimmunoassay based 17-hydroxyprogesterone values of screened population according to birth weight and gestational age

**Table 2:**
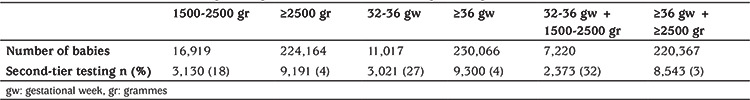
Rate of second-tier testing among babies based on birth weight and gestational weeks

**Table 3:**
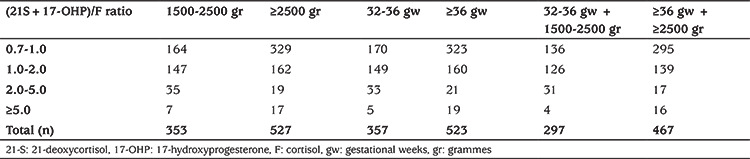
Distribution of babies based on (21-deoxycortisol+17-hydroxyprogesterone)/cortisol ratio adjusted for gestational age and birth weight

**Table 4:**
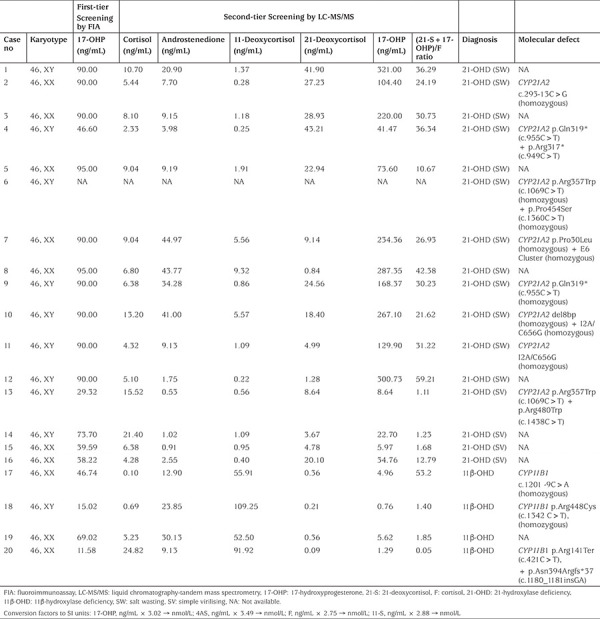
Biochemical and molecular characteristics of congenital adrenal hyperplasia (CAH) patients diagnosed in extended pilot study of neonatal CAH screening in Turkey

**Table 5:**
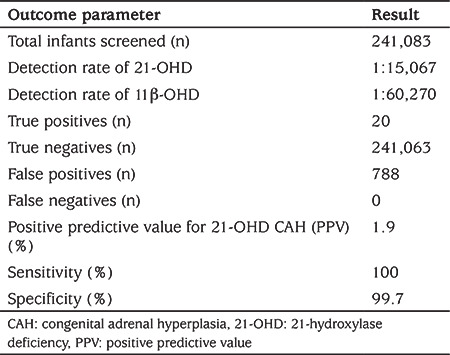
Summary of the results of screening protocol for congenital adrenal hyperplasia in Turkey (March to December, 2018)

## References

[1] Güran T, Tezel B, Gürbüz F, Selver Eklioğlu B, Hatipoğlu N, Kara C, Şimşek E, Çizmecioğlu FM, Ozon A, Baş F, Aydın M, Darendeliler F (2019). Neonatal Screening for Congenital Adrenal Hyperplasia in Turkey: A Pilot Study with 38,935 Infants. J Clin Res Pediatr Endocrinol.

[2] Speiser PW, Arlt W, Auchus RJ, Baskin LS, Conway GS, Merke DP, Meyer-Bahlburg HFL, Miller WL, Murad MH, Oberfield SE, White PC (2018). Congenital Adrenal Hyperplasia Due to Steroid 21-Hydroxylase Deficiency: An Endocrine Society Clinical Practice Guideline. J Clin Endocrinol Metab.

[3] Khattab A, Haider S, Kumar A, Dhawan S, Alam D, Romero R, Burns J, Li D, Estatico J, Rahi S, Fatima S, Alzahrani A, Hafez M, Musa N, Razzghy Azar M, Khaloul N, Gribaa M, Saad A, Charfeddine IB, Bilharinho de Mendonça B, Belgorosky A, Dumic K, Dumic M, Aisenberg J, Kandemir N, Alikasifoglu A, Ozon A, Gonc N, Cheng T, Kuhnle-Krahl U, Cappa M, Holterhus PM, Nour MA, Pacaud D, Holtzman A, Li S, Zaidi M, Yuen T, New MI (2017). Clinical, genetic, and structural basis of congenital adrenal hyperplasia due to 11β-hydroxylase deficiency. Proc Natl Acad Sci U S A.

[4] Hayashi G, Faure C, Brondi MF, Vallejos C, Soares D, Oliveira E, Brito VN, Mendonca BB, Bachega TA (2011). Weight-adjusted neonatal 17OH-progesterone cutoff levels improve the efficiency of newborn screening for congenital adrenal hyperplasia. Arq Bras Endocrinol Metabol.

[5] Sarafoglou K, Banks K, Gaviglio A, Hietala A, McCann M, Thomas W (2012). Comparison of one-tier and two-tier newborn screening metrics for congenital adrenal hyperplasia. Pediatrics.

[6] Steigert M, Schoenle EJ, Biason-Lauber A, Torresani T (2002). High reliability of neonatal screening for congenital adrenal hyperplasia in Switzerland. J Clin Endocrinol Metab.

[7] Chu SY, Tsai WY, Chen LH, Wei ML, Chien YH, Hwu WL (2002). Neonatal screening for congenital adrenal hyperplasia in Taiwan: a pilot study. J Formos Med Assoc.

[8] Silveira EL, dos Santos EP, Bachega TA, van der Linden Nader I, Gross JL, Elnecave RH (2008). The actual incidence of congenital adrenal hyperplasia in Brazil may not be as high as inferred--an estimate based on a public neonatal screening program in the state of Goiás. J Pediatr Endocrinol Metab.

[9] Gruñieiro-Papendieck L, Chiesa A, Mendez V, Prieto L (2008). Neonatal screening for congenital adrenal hyperplasia: experience and results in Argentina. J Pediatr Endocrinol Metab.

[10] van der Linde AAA, Schönbeck Y, van der Kamp HJ, van den Akker ELT, van Albada ME, Boelen A, Finken MJJ, Hannema SE, Hoorweg- Nijman G, Odink RJ, Schielen PCJI, Straetemans S, van Trotsenburg PS, Claahsen-van der Grinten HL, Verkerk PH (2019). Evaluation of the Dutch neonatal screening for congenital adrenal hyperplasia. Arch Dis Child.

[11] Gidlöf S, Wedell A, Guthenberg C, von Döbeln UV, Nordenström A (2014). Nationwide neonatal screening for congenital adrenal hyperplasia in Sweden: a 26-year longitudinal prospective population-based study. JAMA Pediatr.

[12] Pode-Shakked N, Blau A, Pode-Shakked B, Tiosano D, Weintrob N, Eyal O, Zung A, Levy-Khademi F, Tenenbaum-Rakover Y, Zangen D, Gillis D, Pinhas-Hamiel O, Loewenthal N, de Vries L, Landau Z, Rachmiel M, Abu-Libdeh A, Eliakim A, Strich D, Koren I, German A, Sack J, Almashanu S (2019). Combined Gestational Age- and Birth Weight-Adjusted Cutoffs for Newborn Screening of Congenital Adrenal Hyperplasia. J Clin Endocrinol Metab.

